# A novel tailored biliary stent with a controllable cover

**DOI:** 10.1055/a-2719-8097

**Published:** 2025-11-06

**Authors:** Xianrong Hu, Bing Hu, Jun Wu, Qiang Liu, Cui Chen, Ting Zhang, Shuping Wang

**Affiliations:** 1 535219 Department of Gastroenterology and Endoscopy, The Third Affiliated Hospital of Naval Medical University, Shanghai, China

We designed a controllable partially covered SEMS, which provides a more flexible treatment option for patients with malignant biliary stricture. The new design consists of two separate layers ( Fig. 1 ). The outer layer is a short silicone membrane sheath, and the inner layer is a long self-expanding stent, which is installed in one release system that needs to be displaced one by one. In the actual operation process, first slowly release the front-covered sheath to the targeted position. Then, advance the release system inward and displace the uncovered SEMS part inside the covered sheath.

**Fig. 1:**
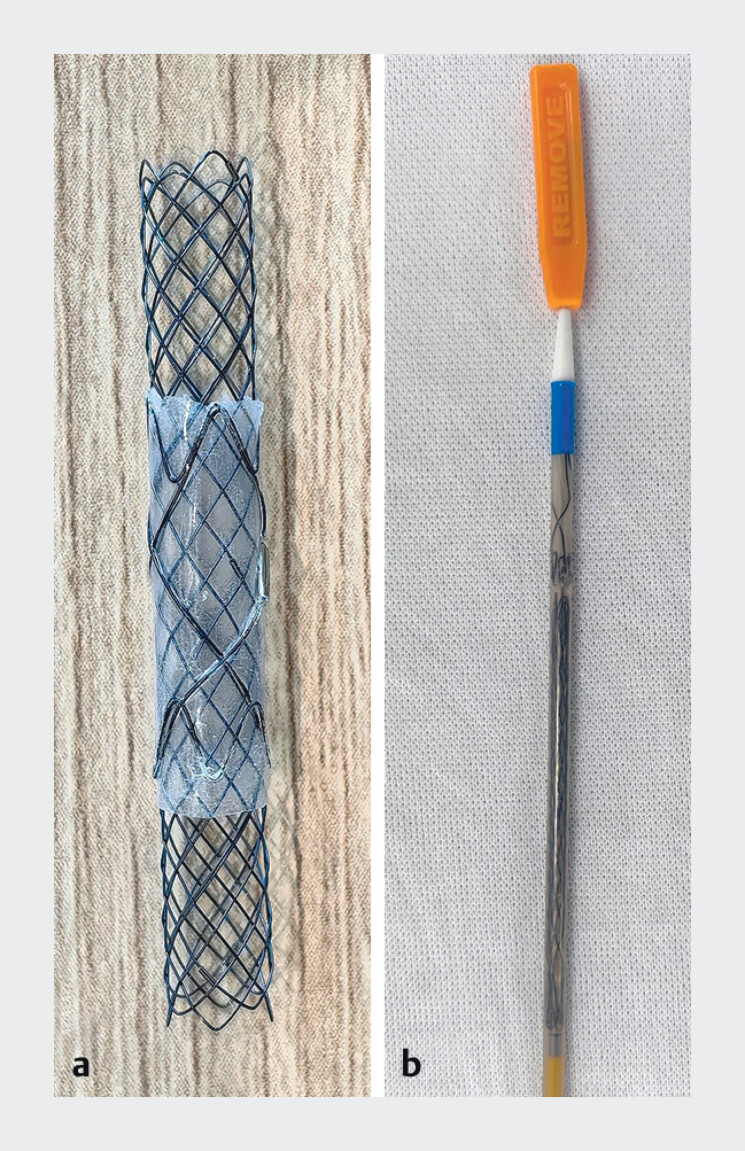
**a** The controllable partially covered SEMS **b** and its delivery system.

In this video ( Video 1 ), we show the release process of this controllable covered SEMS in the bile duct in a porcine model. Firstly, contrast agent was injected to visualize the biliary system, through which we clarified the level of the cystic duct orifice, which was used as a landmark for the placement of the new SEMS ( Fig. 2 ). Based on the landmark guidance, we released the covered sheath first and ensure that its upper edge does not exceed the cystic duct orifice ( Fig. 3 ). Then, we inserted the delivery system and released the internal uncovered SEMS ( Fig. 4 ). At last, we injected some contrast again to confirm that the cystic duct was not covered by the stent ( Fig. 5 ).

The implantation of the controllable partially covered SEMS in a porcine model.Video 1

**Fig. 2:**
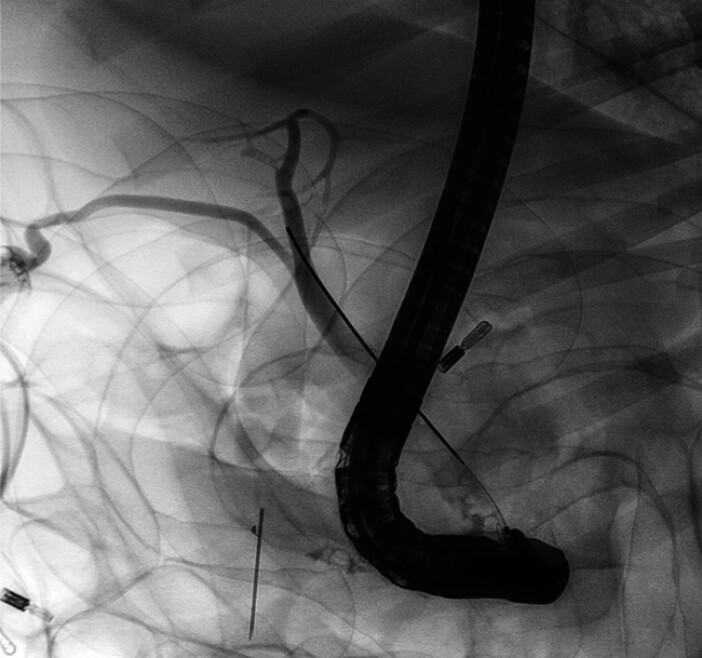
Visualization of the bile ducts and the cystic duct.

**Fig. 3:**
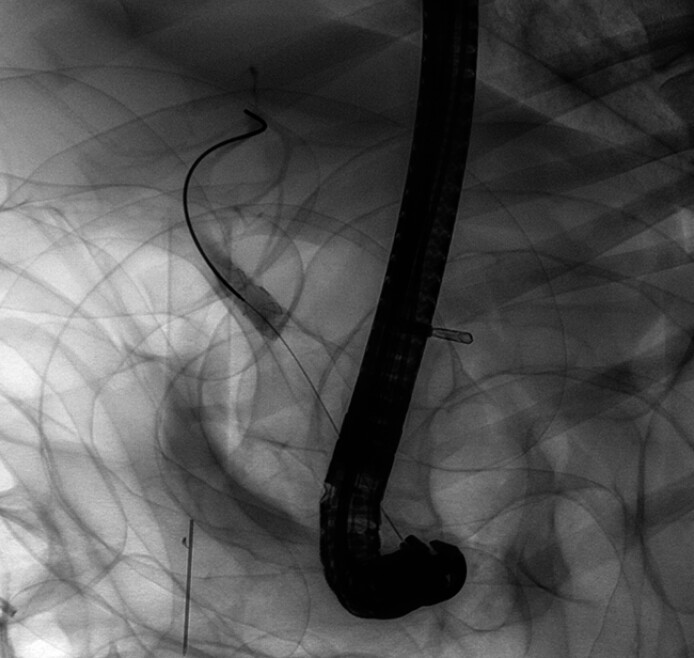
Release the covered sheath below the cystic duct orifice.

**Fig. 4:**
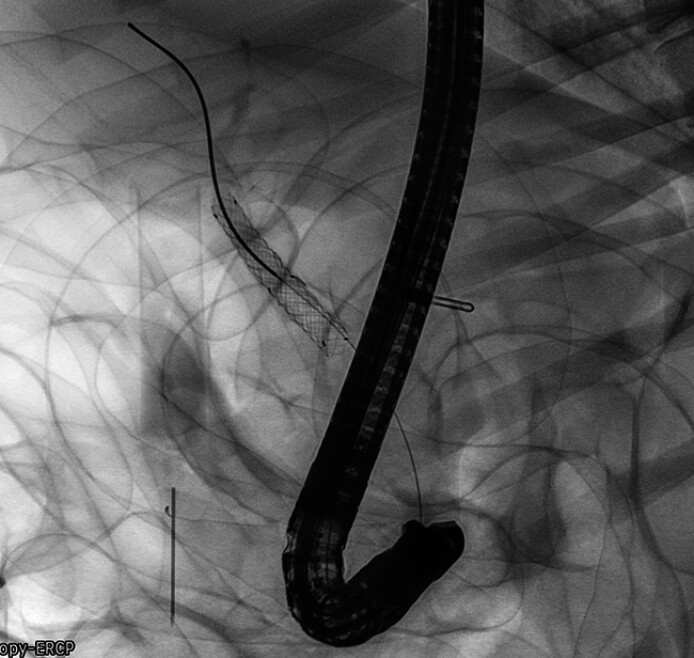
Release the internal uncovered SEMS.

**Fig. 5:**
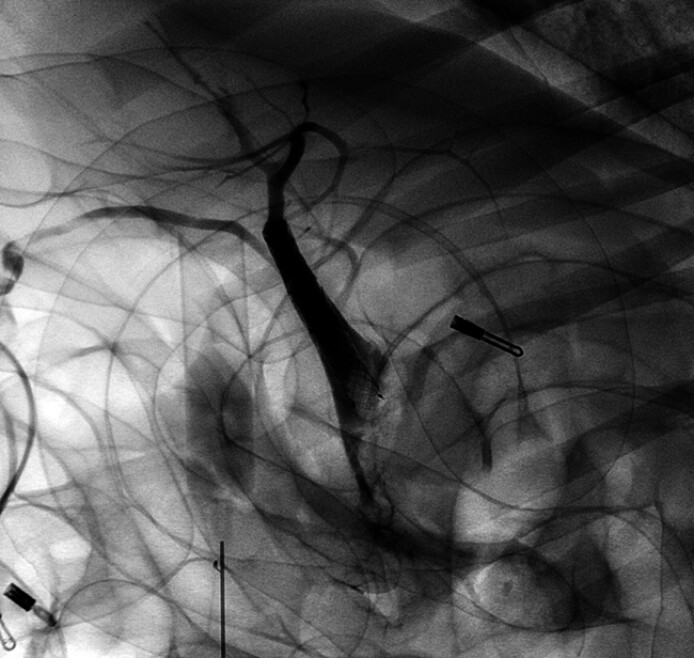
Cholangiography was performed again to show the patent cystic duct.

Fully covered and uncovered SEMS have their own disadvantages 1 . The existing partially covered SEMSs cannot meet the individual needs of patients clinically. In conclusion, this new controllable covered SEMS allows us to accurately perform biliary stenting according to the actual situation, which is expected to improve the treatment effect.

Endoscopy_UCTN_Code_TTT_1AR_2AZ

## Notes

Endoscopy E-Videos https://eref.thieme.de/e-videos
*E-Videos* is an open access online section of the journal *Endoscopy* , reporting on interesting cases and new techniques in gastroenterological endoscopy. All papers include a high-quality video and are published with a Creative Commons CC-BY license. Endoscopy E-Videos qualify for HINARI discounts and waivers and eligibility is automatically checked during the submission process. We grant 100% waivers to articles whose corresponding authors are based in Group A countries and 50% waivers to those who are based in Group B countries as classified by Research4Life (see: https://www.research4life.org/access/eligibility/ ). This section has its own submission website at https://mc.manuscriptcentral.com/e-videos .
